# A novel risk classification system based on the eighth edition of TNM frameworks for esophageal adenocarcinoma patients: A deep learning approach

**DOI:** 10.3389/fonc.2022.887841

**Published:** 2022-12-07

**Authors:** Qiang Shen, Hongyu Chen

**Affiliations:** ^1^ Department of General Surgery, Ningbo No.9 Hospital, Ningbo, Zhejiang, China; ^2^ Department of Thoracic Surgery, Ningbo No.9 Hospital, Ningbo, Zhejiang, China

**Keywords:** deep learning, clinical decision- making, prognosis, prognosis carcinoma, esophageal adenocarcinoma (EAC)

## Abstract

**Objective:**

To develop and validate a deep learning predictive model with better performance in survival estimation of esophageal adenocarcinoma (EAC).

**Method:**

Cases diagnosed between January 2010 and December 2018 were extracted from the Surveillance, Epidemiology, and End Results (SEER) database. A deep learning survival neural network was developed and validated based on 17 variables, including demographic information, clinicopathological characteristics, and treatment details. Based on the total risk score derived from this algorithm, a novel risk classification system was constructed and compared with the 8th edition of the tumor, node, and metastasis (TNM) staging system.

**Results:**

Of 7,764 EAC patients eligible for the study, 6,818 (87.8%) were men and the median (interquartile range, IQR) age was 65 (58–72) years. The deep learning model generated significantly superior predictions to the 8th edition staging system on the test data set (C-index: 0.773 [95% CI, 0.757–0.789] *vs*. 0.683 [95% CI, 0.667–0.699]; *P* < 0.001). Calibration curves revealed that the deep learning model was well calibrated for 1- and 3-year OS, most points almost directly distributing on the 45° line. Decision curve analyses (DCAs) showed that the novel risk classification system exhibited a more significant positive net benefit than the TNM staging system. A user-friendly and precise web-based calculator with a portably executable file was implemented to visualize the deep learning predictive model.

**Conclusion:**

A deep learning predictive model was developed and validated, which possesses more excellent calibration and discrimination abilities in survival prediction of EAC. The novel risk classification system based on the deep learning algorithm may serve as a useful tool in clinical decision making given its easy-to-use and better clinical applicability.

## Introduction

Over the past few decades, the incidence of esophageal adenocarcinoma (EAC) has increased substantially in many Western populations and with most patients diagnosed at advanced stages (1–3). Despite recent advances in multimodality treatment modalities, the prognosis of EAC remains poor, with a dismal overall 5-year survival rate of around 20% (4, 5). The precise risk stratification according to survival outcomes of patients with EAC represents a crucial determinant of treatment (6). The eighth edition of the American Joint Committee on Cancer (AJCC) staging scheme still classifies patients with EAC based on tumor, node, and metastasis (TNM) frameworks, which may tailor limited survival estimations to individuals (7, 8).

It has been widely acknowledged that a variety of potential prognostic factors unaccounted for by the current TNM stage groupings (such as age, gender, tumor differentiation, and treatment choices) could significantly contribute to individualized predictions of survival (9–11). As such, studies with different methods to improve the accuracy of prognostication have been implemented. However, the majority of these studies generated prognostic tools based on the Cox proportional hazards (CPH) model, which hardly handle potentially non-linear correlations in survival analyses. As a result, the discriminative ability of these tools may be just passable (12–14).

With the rapid progress in artificial intelligence (AI) recently, deep learning is a promising solution to this problem (15). As the state-of-the-art algorithm, deep learning allows a prognostic network to automatically discover the potentially non-linear relationships with the use of multiple neural layers (16). In application, these networks, especially combined with a large-scale cohort, have shown great potential in many prognostic studies, such as lung cancer and breast cancer (17, 18). However, to date, studies taking advantage of the deep learning algorithm are absent in the prognosis of EAC.

Using a large population-based cancer database, the present study was designed to develop a deep learning survival neural network with better predictive performance for patients with EAC. With this neural network, we also attempt to construct a novel and more precise risk classification system based on the 8th edition of TNM frameworks.

## Methods

### Patient selection and data preparation

**Figure 1:**
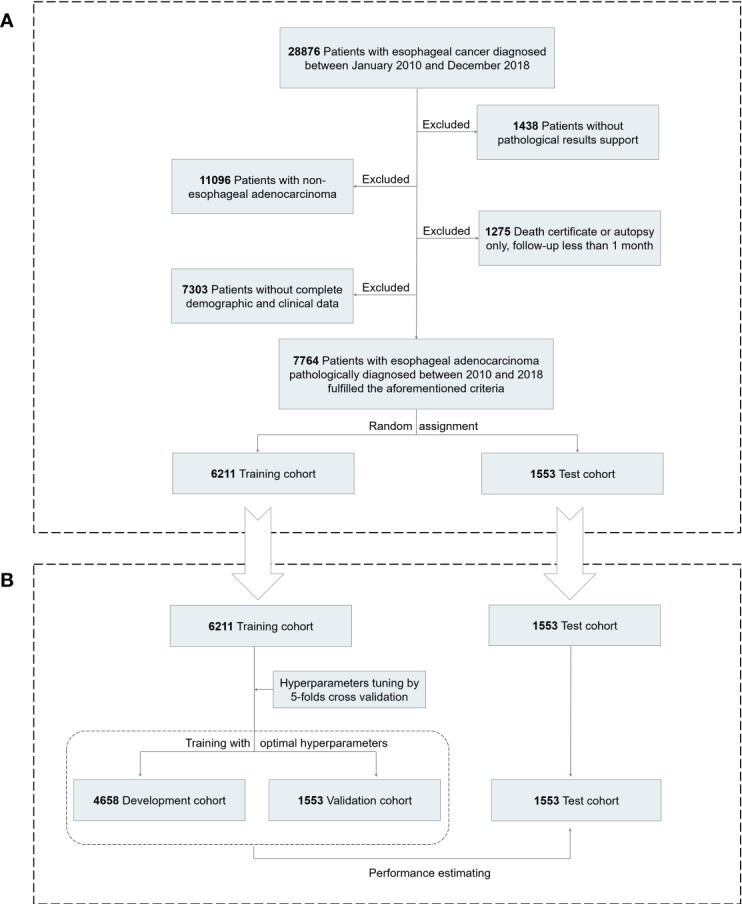
Analytical framework for survival prediction. **(A)** Flowchart showing derivation of the training and test cohorts. **(B)** A detailed pipeline to develop, validate, and test the deep learning model.

The current study used data from a prospectively maintained and nationwide cancer database, the Surveillance, Epidemiology, and End Results (SEER) database. All primary patients with EAC pathologically diagnosed between January 2010 and December 2018 were initially considered eligible for our study. We collected the demographic information of patients (age, gender, race, and marital status), clinicopathological characteristics (tumor [T] stage, nodal [N] stage, metastasis [M] stage, metastatic site [bone, brain, liver, lung], histologic grade, tumor location and size), and treatment choices (surgery, chemotherapy, and radiation), with a total of 17 potentially influencing variables. Excluded were cases identified by autopsy or death certificate only, follow-up less than 1 month, and those who lacked any of the included features as mentioned above. Finally, a total of 7,764 cases were selected for further analyses, randomly divided into the training and test cohorts with the ratio of 8:2 (**Figure 1A**). This study was deemed exempt from the institutional review board (IRB), since any identifiable information of patients in this database is unavailable. All methods were carried out in accordance with relevant guidelines and regulations.

In this study, marital status was reclassified as single, married, divorced/separated, and widowed. Since only 38 cases were unmarried or domestic partners, we reassigned them as single. The TNM stage was also reclassified to generate a uniform dataset according to the eighth edition of the AJCC staging system. Thus, for patients diagnosed before 2018, we manually translated 7th-edition stages into their corresponding 8th-edition stages. Then, all T stages were redefined into T1, T2, T3, T4, and TX. For instance, T1 stages were consisting of T1a and T1b. Similarly, all N stages were transformed into N0, N1, N2, N3, and NX.

### Deep learning survival neural network

In this study, we develop the deep learning survival neural network with a method referred to as DeepSurv, which was designed by Katzman (**Figure 1B**) (19). In brief, the deep learning model contained a fully connected feed-forward neural network structure with a single output node to calculate the survival risks of patients using the negative log-partial likelihood function. Firstly, all numerical covariates were standardized and categorical features were transformed as dummy variables when tuning. In the present study, all 17 variables described above were included. A detailed dataset description is presented in **Table 1**. Next, the grid search method was adopted to select the optimal hyperparameters of DeepSurv. To minimize model overfitting, the optimal hyperparameters were determined according to the least validation loss from fivefold cross validation. Then, based on the optimal hyperparameters, we developed a deep learning survival neural network comprising three hidden layers, each of which has 40 neurons. The selected optimal hyperparameters were as follows: the dropout rate was 0.3, the learning rate was 0.002, the batch size was 200, and the optimizer was Adam. Lastly, to confirm the robustness of our neural network, we attempted to develop the model with other different random seeds. As shown in **Supplementary Figure S1**, the discriminative ability of the network was relatively robust. The current study was in line with the TRIPOD guideline (Supplementary Material: Tripod-Checklist-Prediction-Model-Development).

**Table 1:** Patients’ demographic and clinicopathological characteristics.

Characteristic	All patients (n = 7,764)	No. of patients (%)		*P* value
		Training cohort (n = 6,211)	Test cohort (n = 1,553)	
Age, median (IQR), years	65 (58-72)	65 (58-72)	65 (58-73)	0.729
Sex				0.066
Female	946 (12.2)	778 (12.5)	168 (10.8)	
Male	6,818 (87.8)	5,433 (87.5)	1,385 (89.2)	
Race				0.931
White	7,323 (94.3)	5,857 (94.3)	1,466 (94.4)	
Black	200 (2.6)	162 (2.6)	38 (2.4)	
Others	241 (3.1)	192 (3.1)	49 (3.2)	
Marital status				0.370
Single/unmarried	1,271 (16.4)	1,023 (16.5)	248 (16.0)	
Married	4,957 (63.8)	3,984 (64.1)	973 (62.7)	
Divorced/separated	943 (12.1)	738 (11.9)	205 (13.2)	
Widowed	593 (7.6)	466 (7.5)	127 (8.2)	
Stage				0.437
I	1,391 (17.9)	1,093 (17.6)	298 (19.2)	
II	1,151 (14.8)	915 (14.7)	236 (15.2)	
III	2,459 (31.7)	1,980 (31.9)	479 (30.8)	
IV	2,763 (35.6)	2,223 (35.8)	540 (34.8)	
T stage				0.451
T1	1,934 (24.9)	1,531 (24.6)	403 (25.9)	
T2	950 (12.2)	765 (12.3)	185 (11.9)	
T3	3,380 (43.5)	2,696 (43.4)	684 (44.0)	
T4	652 (8.4)	522 (8.4)	130 (8.4)	
TX	848 (10.9)	697 (11.2)	151 (9.7)	
N stage				0.278
N0	2,877 (37.1)	2,273 (36.6)	604 (38.9)	
N1	3,349 (43.1)	2,686 (43.2)	663 (42.7)	
N2	944 (12.2)	765 (12.3)	179 (11.5)	
N3	389 (5.0)	314 (5.1)	75 (4.8)	
NX	205 (2.6)	173 (2.8)	32 (2.1)	
M stage				0.392
M0	5,289 (68.1)	4,217 (67.9)	1,072 (69.0)	
M1	2,475 (31.9)	1,994 (32.1)	481 (31.0)	
Tumor size, median (IQR), mm	44 (28-60)	44 (28-60)	44 (29-60)	0.942
Tumor differentiation				0.636
Well	1,432 (18.4)	1,161 (18.7)	271 (17.5)	
Moderate	2,608 (33.6)	2,071 (33.3)	537 (34.6)	
Poor	2,955 (38.1)	2,367 (38.1)	588 (37.9)	
Unknown	769 (9.9)	612 (9.9)	157 (10.1)	
Tumor location				0.644
Upper	78 (1.0)	57 (0.9)	21 (1.4)	
Middle	450 (5.8)	363 (5.8)	87 (5.6)	
Lower	6,445 (83.0)	5,160 (83.1)	1,285 (82.7)	
Overlap	322 (4.1)	257 (4.1)	65 (4.2)	
NOS	469 (6.0)	374 (6.0)	95 (6.1)	
Metastasis at bone				0.383
No	7,136 (91.9)	5,717 (92.0)	1,419 (91.4)	
Yes	628 (8.1)	494 (8.0)	134 (8.6)	
Metastasis at brain				0.855
No	7,569 (97.5)	6,054 (97.5)	1,515 (97.6)	
Yes	195 (2.5)	157 (2.5)	38 (2.4)	
Metastasis at liver				0.094
No	6,547 (84.3)	5,216 (84.0)	1,331 (85.7)	
Yes	1,217 (15.7)	995 (16.0)	222 (14.3)	
Metastasis at lung				0.646
No	7,146 (92.0)	5,721 (92.1)	1,425 (91.8)	
Yes	618 (8.0)	490 (7.9)	128 (8.2)	
Surgery				0.498
No	4,470 (57.6)	3,594 (57.9)	876 (56.4)	
Partial	825 (10.6)	661 (10.6)	164 (10.6)	
Total	2,469 (31.8)	1,956 (31.5)	513 (33.0)	
Radiation				0.074
None/unknown	2,754 (35.5)	2,173 (35.0)	581 (37.4)	
Yes	5,010 (64.5)	4,038 (65.0)	972 (62.6)	
Chemotherapy				0.398
None/unknown	1,960 (25.2)	1,555 (25.0)	405 (26.1)	
Yes	5,804 (74.8)	4,656 (75.0)	1,148 (73.9)	

IQR, interquartile range.

### Statistical analysis

The primary end point was overall survival (OS), which was calculated from the date of diagnosis to the time of death from any cause or last follow-up observation. Continuous variables are presented as medians with interquartile range (IQR), whereas categorical variables are presented as frequencies with percentages. The training and test cohorts were compared with the Mann–Whitney test for continuous variables or the chi-square test in case of categorical variables. In addition to the neural network, a CPH model was also constructed by applying a backward approach based on the Akaike information criterion (AIC). Harrell’s concordance index (C-index) and calibration curves were utilized to evaluate the predictive performance, and compared between the proposed models and the 8th edition of the AJCC staging system.

Then, we further derived the total risk score from the neural network. According to the quartile of the total risk score, patients with EAC were divided into four groups to construct a novel risk classification system. Survival curves were plotted with the Kaplan–Meier method and compared using the log-rank test. Decision curve analyses (DCAs) were performed to evaluate the clinical utility and compared between the novel risk classification system and the 8th edition of the AJCC staging system. With pandas and Scikit-learn packages utilized for the treatment of data, the deep learning survival neural network was developed on the PyTorch framework. Other statistical analyses were performed with R software (Edition of 4.0.2, R Foundation, Vienna, Austria); a 2-sided *P*-value < 0.05 was considered statistically significant.

## Results

### Patient demographics and characteristics

A total of 7,764 patients with EAC were eligible for the study (median [IQR] age, 65 [58–72] years; 6,818 [87.8%] men), of which 6,211 were assigned to the training cohort, whereas 1,553 were assigned to the test cohort (**Figure 1**). The training and test cohorts showed similar distributions in demographic and clinical characteristics (**Table 1**). More than 90% of EAC patients were white, with the majority diagnosed at advanced stages (5,222 [67.3%]). Median follow-up was 50 months (95% CI, 48–52 months). There were 5,050 patients (65.0%) who died during the follow-up period, of which 4,418 (56.9%) were deaths from EAC.

### Calibration and validation of the deep learning model in the test cohort

We compared the discriminative ability of the deep learning model to that of the 8th edition of the TNM staging system in the test cohort (**Table 2**). The deep learning model generated significantly superior predictions to the 8th-edition staging system (C-index: 0.773 [95% CI, 0.757–0.789] *vs*. 0.683 [95% CI, 0.667–0.699]; *P* < 0.001). Similarly, the C-index of the deep learning model was also significantly superior to that of the CPH model (C-index for CPH was 0.748 [95% CI, 0.732–0.764]; *P* < 0.001). Calibration curves revealed that the deep learning model was best calibrated, with the superb agreement between the predicted probabilities and the actual outcomes of all patients with EAC for 1- and 3-year OS, most points almost directly distributing on the 45° line (**Supplementary Figure S2**).

**Table 2:** Comparison of C-indices between the proposed models and the 8th edition of the AJCC staging system.

Characteristic	Deep learning	CPH	8th TNM stage
C-index (95% CI)	0.773 (0.757-0.789)	0.748 (0.732-0.764)	0.683 (0.667-0.699)
*P* value^a^	–	<0.001	<0.001

CPH, Cox proportional hazards model.

^a^C-indices of CPH and the 8th edition of the TNM staging system compared with the deep learning model.

### Model visualization and establishment of a novel risk classification system

**Figure 2:**
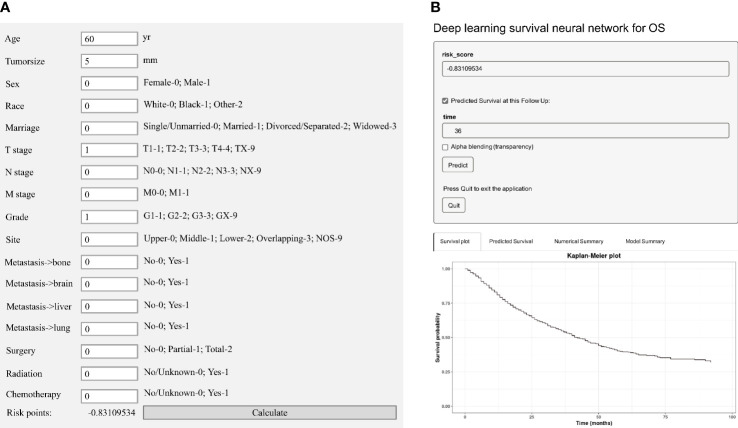
Model visualization of the deep learning predictive model. **(A)** A portably executable file to calculate the total risk score according to the input characteristics. **(B)** A web-based calculator for overall survival (OS) estimation in patients with EAC.

We established an easy-to-use and precise web-based calculator (https://web-calculator.shinyapps.io/DynNomapp/) with a portably executable file (https://pan.baidu.com/s/1DUU-x6XbpYHfmf1Kf1dFNA?pwd=1234). The total risk score for each patient would be calculated automatically by the application according to the input characteristics (**Figure 2A**). Then, the web-based calculator would conveniently provide the precise predicted probability of OS based on the total risk score (**Figure 2B**).

**Figure 3:**
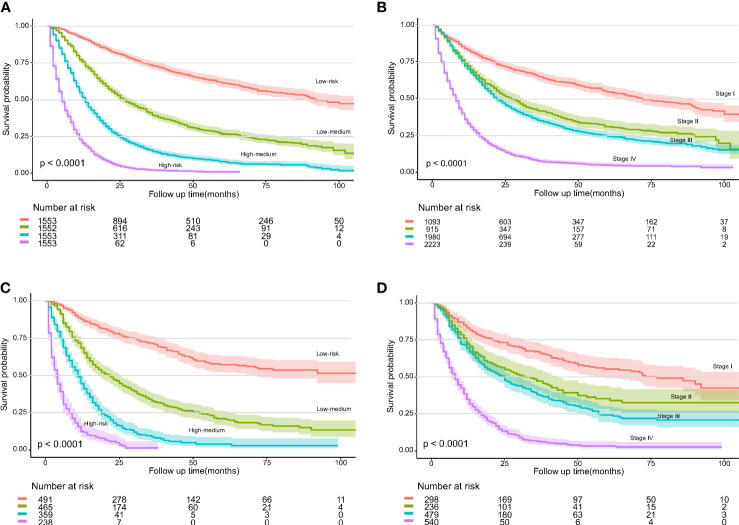
Kaplan–Meier curves for overall survival (OS) according to the different staging systems. The novel risk classification system in the training cohort **(A)** and test cohort **(C)**. The eighth edition of the TNM staging system in the training cohort **(B)** and test cohort **(D)**.

Next, according to the quartile of the total risk score, we attempted to assign patients with EAC into four risk groups to construct a novel risk classification system. Compared with the 8th edition of the TNM staging system, the Kaplan–Meier curves showed that the novel risk classification system seemed to better distinguish patients with different risks in both the training and test cohorts (**Figure 3**). Further, DCAs also showed that the novel risk classification system exhibited a more significant positive net benefit than the 8th edition of the TNM staging system (**Supplementary Figure S3**).

## Discussion

Overall, our pilot study was designed to construct a deep learning neural network in survival prediction for newly diagnosed patients with EAC. This large-scale study demonstrated that the deep learning model possessed significantly superior predictive performance than the traditional TNM staging system. Moreover, we succeeded to construct a novel and more precise risk classification system based on the 8th edition of the TNM frameworks, which may potentially help clinicians in clinical decision making.

During the past few years, EAC has experienced a dramatic increase in incidence and surpassed esophageal squamous cell carcinoma (ESCC) in many Western countries, including the United States (20, 21). It is estimated that the incidence of EAC will continuously increase up to 2030, which certainly imposes economic burdens (22). As such, evidence-based treatment opinions on optimal strategy recommending are crucial for reducing such burdens. The eighth edition of the TNM staging scheme was the most well-validated prognostic indicator for EAC. However, patients within the same stage cohort vary widely in the survival rate (12). Other potential prognostic factors, which are not included in these TNM stage groupings, could affect the outcome, from patient-specific factors such as age and gender to tumor-related information such as grade and tumor location (23). Incorporating these various characteristics of each EAC patient to provide an accurate prediction of prognosis is challenging in the absence of easy-to-use and comprehensive predictive models.

Previous research has reported a variety of predictive tools based on linear CPH for survival prediction in patients with EAC (12–14). By integrating other potentially independent factors with TNM stage, these linear models to some extent derive more precise risk stratification for patients with EAC. However, CPH assume that a patient’s log-risk of death is a simplistic linear combination of some observed covariates, which cannot handle the non-linear relationships and failed to include some potentially colinear but important prognostic factors (17, 24).

AI has been more and more popular in various disciplines for its ability to mimic an intelligent human mind’s cognitive behavior (22). In gastroenterology, AI-based technologies, which are characterized by deep learning as state-of-the-art algorithms, have been already applied in many aspects, such as to diagnose dysplasia in Barrett’s esophagus (BE), to identify *Helicobacter pylori* in the upper gastrointestinal (UGI) tract, and other diagnostic applications (25–27). Nevertheless, few studies have focused on its performance in the prediction of survival for EAC patients, to our knowledge. The present study is the first to develop a more accurate predictive tool for EAC patients using a novel deep learning model, DeepSurv. In this study, a deep learning survival neural network integrating the demographic information of patients, characteristics of tumor extent, and type of treatment was established and validated, with calibration curves revealing excellent agreement between the predicted and actual OS probability. Moreover, the results showed that this neural network significantly outperformed use of the TNM staging alone, as well as the CPH model, which provide additional evidence of the superior predictive accuracy of deep learning models over conventional approaches.

In addition, according to the quartile of the total risk score derived from the deep learning model, we also attempted to assign EAC patients into four risk groups to construct a novel risk classification system. The DCAs showed that a more significant positive net benefit was observed in the novel risk classification system than the TNM staging system. Given that an easy-to-use and precise web-based calculator was implemented, we do believe this novel risk classification system would have widespread acceptance and play a big role in clinical decision making.

Despite these strengths, it is important to recognize several limitations of the present study. Firstly, it is regrettable that the SEER database could not provide information on lifestyle habits and overall comorbidity, more information about peritoneal metastases, and biomarkers in the laboratory, which may further improve the predictive accuracy (28). Secondly, we also acknowledge it hard to interpret how the deep learning model works because the process of predictions is much like black boxes. Thirdly, although a more precise risk classification system was established with utilizing a large population-based US database, a creditable validation in a non-US population is still warranted.

## Conclusion

The present study is the first, to our knowledge, to construct and validate a deep learning model, which possesses more excellent calibration and discrimination abilities in survival prediction of EAC. This novel risk classification system may serve as a useful tool in clinical decision making given its easy-to-use clinical applicability. Further creditable studies in a non-US population are warranted to validate our deep learning model.

## Data availability statement

Publicly available datasets were analyzed in this study. This data can be found here: https://seer.cancer.gov/.

## Author contributions

Dr QS had full access to all of the data in the study and takes responsibility for the integrity of the data and the accuracy of the data analysis. Concept and design: QS, HC. Acquisition, analysis, or interpretation of data: QS, HC. Statistical analysis: QS. Drafting of the manuscript: QS. Supervision: HC. All authors contributed to the article and approved the submitted version.

## Supplementary material

Supplementary Figure 1 The discriminative ability of the deep learning network in the test cohort with different random seeds.

Supplementary Figure 2 Calibration curves for overall survival (OS) for the deep learning (DL) model, Cox proportional hazards (CPH) model and TNM staging system. DL at 1- year **(A)** and 3- year **(B)**; CPH at 1- year **(C)** and 3- year **(D)**; TNM at 1- year **(E)** and 3- year **(F)**.

Supplementary Figure 3 Decision curve analyses (DCAs) demonstrating the net benefits associated with the use of two different staging systems. **(A)** In the training cohort; **(B)** In the test cohort.
