# Supplementary figures and images for: A novel risk classification system based on the eighth edition of TNM frameworks for esophageal adenocarcinoma patients: A deep learning approach
Source: Front Oncol. 2022 Dec 7;12:887841. doi: 10.3389/fonc.2022.887841 (PMC9768177; doi:10.3389/fonc.2022.887841)

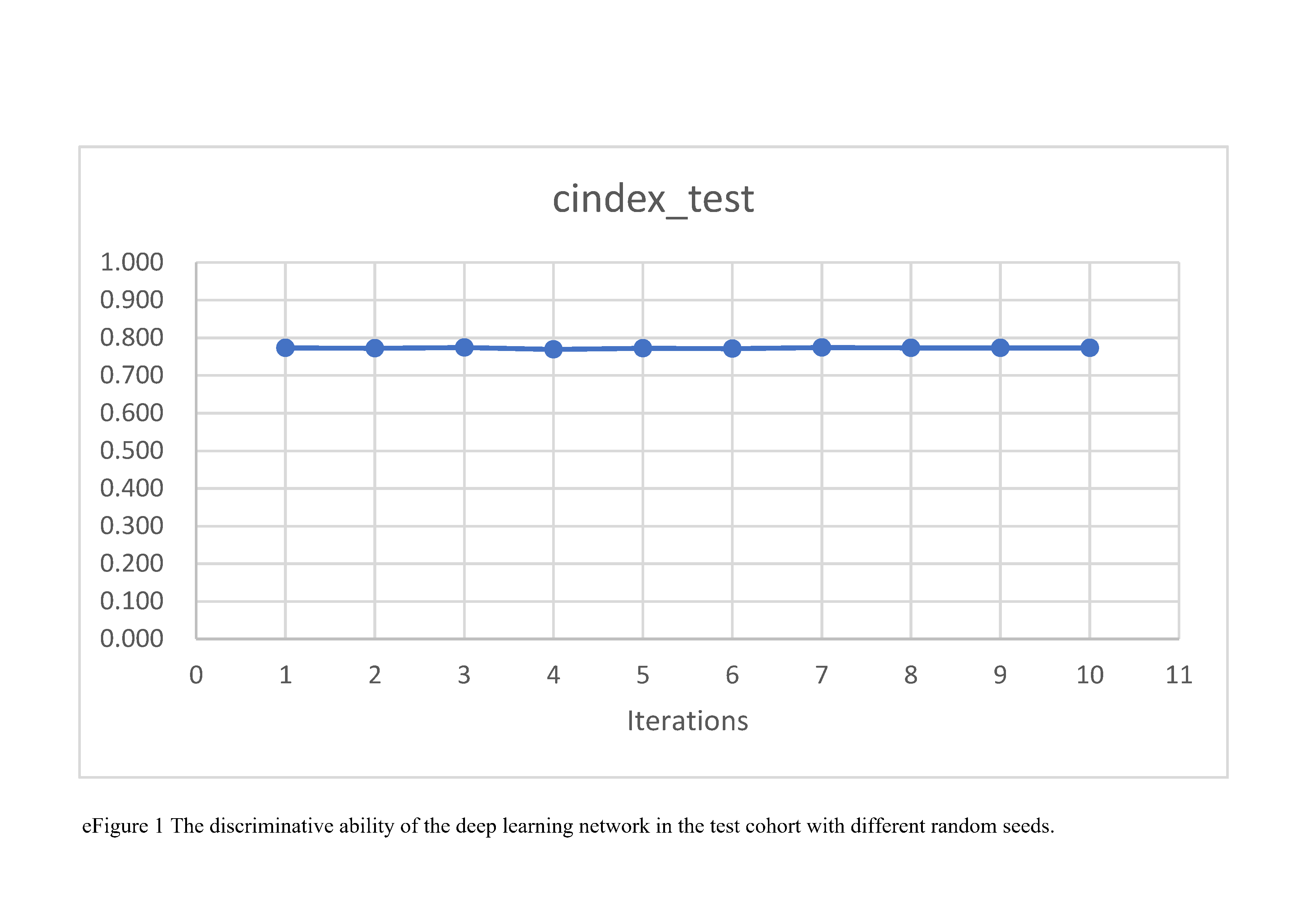

Supplement: Supplementary Figure 1 — The discriminative ability of the deep learning network in the test cohort with different random seeds. [file Image_1.tiff]

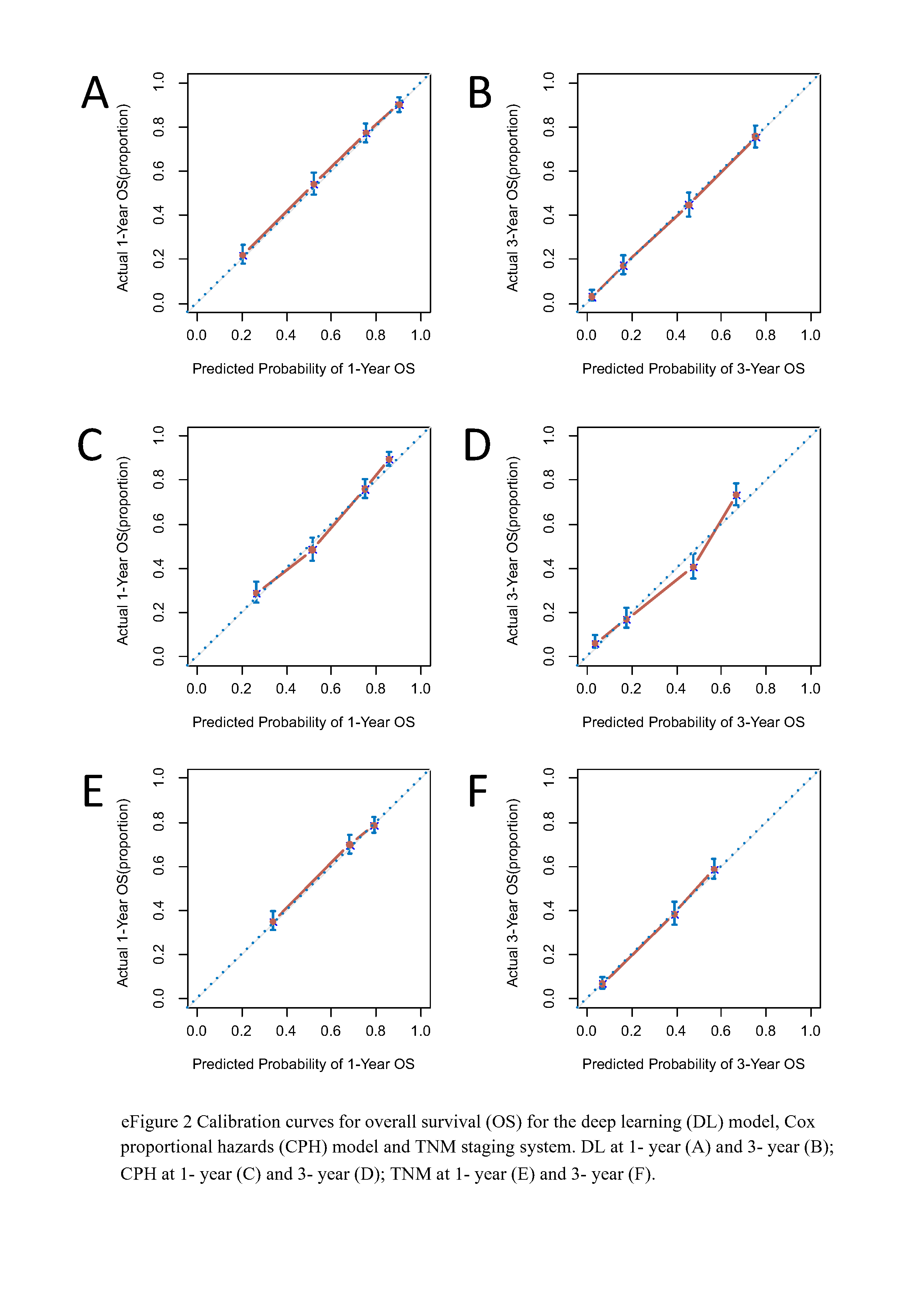

Supplement: Supplementary Figure 2 — Calibration curves for overall survival (OS) for the deep learning (DL) model, Cox proportional hazards (CPH) model and TNM staging system. DL at 1- year (A) and 3- year (B); CPH at 1- year (C) and 3- year (D); TNM at 1- year (E) and 3- year (F). [file Image_2.tiff]

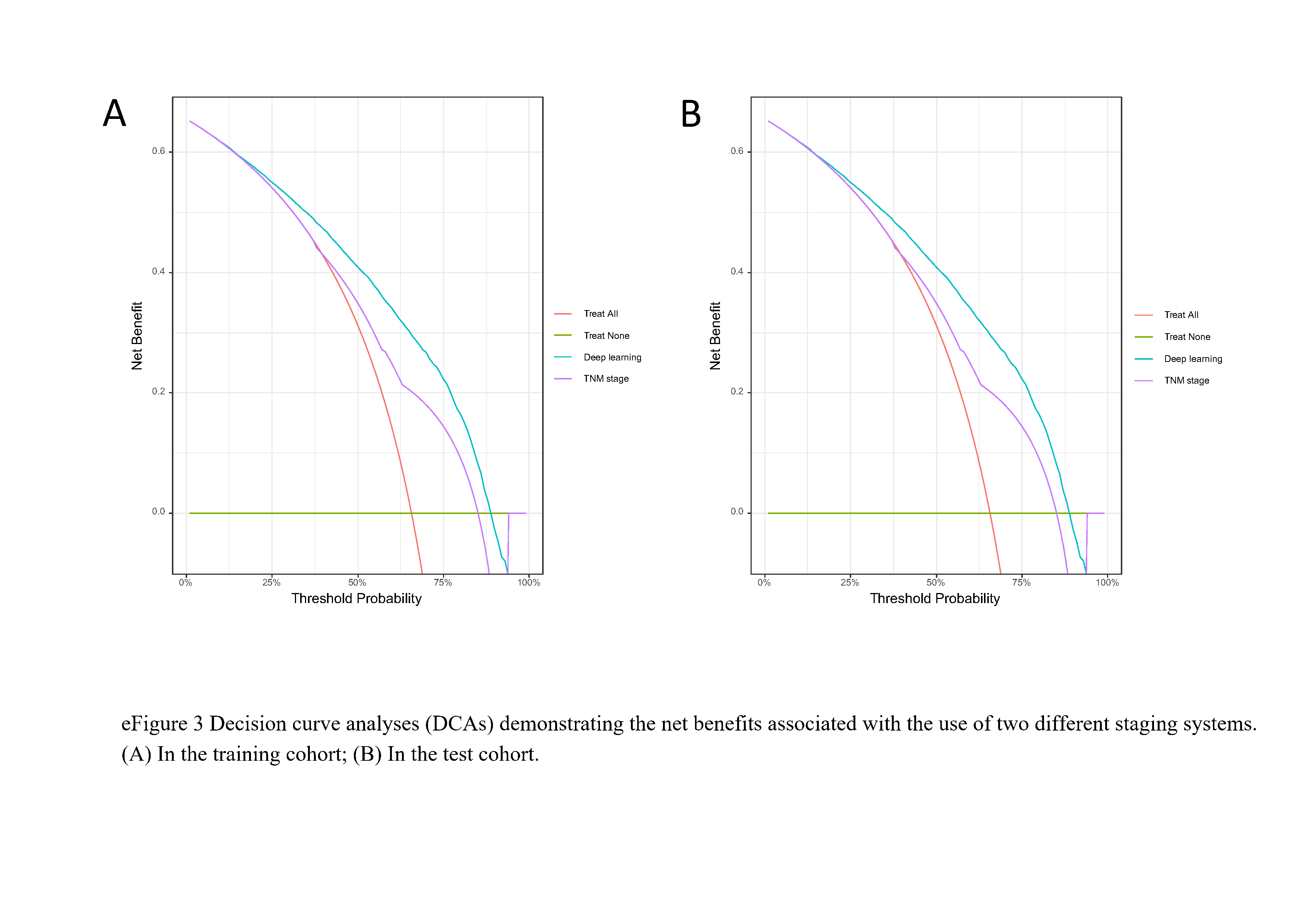

Supplement: Supplementary Figure 3 — Decision curve analyses (DCAs) demonstrating the net benefits associated with the use of two different staging systems. (A) In the training cohort; (B) In the test cohort. [file Image_3.tiff]
